# SOURCES OF VACCINE INFORMATION: MISGIVINGS AND TRUST ISSUES AMONG OLDER ADULTS IN NORTH DAKOTA

**DOI:** 10.1093/geroni/igad104.3597

**Published:** 2023-12-21

**Authors:** Andrea Huseth-Zosel, Heather Fuller

**Affiliations:** North Dakota State University, Fargo, North Dakota, United States; North Dakota State University, Fargo, North Dakota, United States

## Abstract

Older adults consistently have relatively high vaccination rates. What is not fully understood is how trust in immunization information sources impacts vaccine decision-making among older adults. This study examines older adults’ perceptions and decision-making processes for vaccines such as influenza, pneumonia, shingles, and COVID-19. Older adults (65+) in North Dakota (N=51) who participated in a larger survey study on vaccine behaviors voluntarily agreed to participate in a follow-up phone interview (November 2022-January 2023). Qualitative, semi-structured phone interviews focused on access, knowledge, and decision about vaccines. Interview transcripts were inductively coded for emergent themes related to trust. Three themes were identified: implicit trust in information sources, uncertainty who to trust for vaccine information, and regaining trust in information sources. Implicit trust focused on familiarity with sources of information (such as inherently trusting family members), source expertise (such as doctor recommendations), and reliability of sources (including if the source always provides accurate information). The second theme, uncertainty who to trust, stressed confusion over source inconsistency, such as changing positions on vaccines, or having opposing sources that both seem to be trustworthy. The final theme, regaining trust in information sources, highlighted if and how trust in a source of vaccine information could be recovered, including transparency, sincerity, and verifying immunization information through obtaining a second opinion. These findings contribute to the understanding of vaccine decision-making among older adults and provide focus areas for interventions to address lack of trust in information sources and improve immunization rates for this population.

